# Inhibition Effect of STING Agonist SR717 on PRRSV Replication

**DOI:** 10.3390/v16091373

**Published:** 2024-08-29

**Authors:** Xuanying Si, Xiaoge Wang, Hongju Wu, Zhiwei Yan, Longqi You, Geng Liu, Mao Cai, Angke Zhang, Juncheng Liang, Guoyu Yang, Chen Yao, Yongkun Du

**Affiliations:** 1 National International Joint Research Center for Animal Immunology, School of Animal Medicine, Henan Agricultural University, Zhengzhou 450046, China; xuanying1217@163.com (X.S.); 17812168797@163.com (X.W.); wuhongjuvip@163.com (H.W.); yzw2234615939@163.com (Z.Y.); yyy01024641@163.com (L.Y.); liugeng0707@163.com (G.L.); caimao20010511@163.com (M.C.); zhangangke1112@126.com (A.Z.); ljc17790623099@163.com (J.L.); haubiochem@163.com (G.Y.); 2 Key Laboratory of Animal Pathogenesis and Biosafety, Ministry of Education, School of Animal Medicine, Henan Agricultural University, Zhengzhou 450046, China

**Keywords:** PRRSV, SR717, viral life cycle, STING, antiviral

## Abstract

The porcine reproductive and respiratory syndrome virus (PRRSV) belongs to the Arteriviridae family and is a single-stranded, positively stranded RNA virus. The currently available PRRSV vaccines are mainly inactivated and attenuated vaccines, yet none of the commercial vaccines can provide comprehensive, long-lasting, and effective protection against PRRSV. SR717 is a pyridazine-3-carboxamide compound, which is commonly used as a non-nucleoside STING agonist with antitumor and antiviral activities. Nevertheless, there is no evidence that SR717 has any antiviral effects against PRRSV. In this study, a dose-dependent inhibitory effect of SR717 was observed against numerous strains of PRRSV using qRT-PCR, IFA, and WB methods. Furthermore, SR717 was found to stimulate the production of anti-viral molecules and trigger the activation of the signaling cascade known as the stimulator of interferon genes (STING) pathway, which contributed to hindering the reproduction of viruses by a certain margin. Collectively, these results indicate that SR717 is capable of inhibiting PRRSV infection in vitro and may have potential as an antiviral drug against PRRSV.

## 1. Introduction

The first documented case of porcine reproductive and respiratory syndrome (PRRS) in the United States was reported in the late 1980s, followed by the discovery of PRRSV in Europe in the late 1990s, and the isolation of the first PRRSV strain in the Netherlands, which was formally named porcine reproductive and respiratory syndrome (PRRS) in 1992 at the First International SIRS Symposium in Minnesota, USA [1]. Porcine reproductive and respiratory syndrome, often abbreviated as PRRS, is a swift-spreading, infectious viral illness that affects pigs. It is caused by the porcine reproductive and respiratory syndrome virus, or PRRSV for short. PRRSV is a virulent virus with pigs as the exclusive host, belonging to the family Arteritis viridae, and is a single-stranded RNA virus [2]. Among all ages of pigs, the disease is associated with a high rate of mortality and morbidity. It can cause a range of symptoms, including abortion, premature birth, stillbirth, and fetal mummification in sows, as well as being able to cause respiratory distress in piglets. Secondary pathogenic infections are often complicated by the infection itself [3]. The rapidity of genomic mutation and the high frequency of new strains of PRRSV present significant challenges to the prevention and control of PRRSV, and the complete eradication of the disease worldwide remains difficult, as the available commercial vaccines are unable to produce comprehensive and durable control effects [4,5,6].

Invasion by PRRSV can provoke an immune response both natural and humoral. Immune function is disrupted when the lymphocyte population is disrupted by PRRSV infection. However, the infection does not have a significant impact on lymphocyte differentiation or maturation. The natural immune response represents the more conservative immune response mechanism, and the natural immune response is the first line of defense for pathogens invading the organism, mainly including skin, saliva, macrophages, dendritic cells, natural killer cells, complements, etc. [7]. Natural immunity is characterized by rapid response, a large area of action, non-specificity, and non-memory. The natural immune system exerts its action mainly through the recognition of PPAR by PRR. In addition, the natural immune system can also act on foreign pathogens by secreting cytokines, as well as on foreign pathogens by natural killer cells. Studies have demonstrated that Marc-145 cells and PAMs can inhibit the production of PRRSV infection cytokines. To date, it has been reported that the nsp4 protein, nsp1β protein, nsp2 protein, nsp1α protein, nsp11 protein, and N protein of PRRSV have been identified to act as antagonists of IFN expression. The invasion of cells by PRRSV activates proteins such as NF-κB, which in turn triggers the production of cytokines such as IL-6, IL-8, and IL-1β [8,9].

It has been demonstrated that SR717 acts as a stimulator of the STING pathway [10]. It has also been demonstrated that SR717 is capable of eliciting anti-tumor immune responses through the promotion of CD8+ T cell and natural killer cell maturation, cytokine secretion, and the induction of local innate immune responses [11]. SR717 not only cranks up the production of proteins linked to the STING signaling pathway but also gives a significant boost to the expression of cytokine genes. Furthermore, SR717 has been shown to have no significant adverse effects on the body’s blood biochemical indicators or major organs [12,13].

The TMEM173 gene encodes STING (MPYS or MITA), a transmembrane protein in the endoplasmic reticulum. In human cells, the STING protein is composed of 379 amino acids and is structured by an N-terminal transmembrane domain and a C-terminal domain [12,14]. The complete STING signaling pathway comprises the cGAS-cGAMP-STING cascade and downstream proteins. A monomer of cGAS exists in various parts of the cell. cGAS contains a nucleotide transferase structural domain and two DNA-binding structural domains [15,16]. Upon the recognition of viral DNA, it catalyzes the generation of the second messenger cGAMP from ATP and GTP [17,18]. This process is initiated by the interaction of cGAMP with STING, which then undergoes a conformational change. STING is capable of recognizing exogenous DNA as well as localizing cytoplasmic DNA and is able to make immune responses innate following activation [19,20,21]. Two key processes facilitate the activation of STING: palmitoylation of STING and phosphorylation of STING, which interacts with IRF3 and phosphorylates IRF3. This results in the production of cytokines for the antiviral action, thus enabling the body’s natural antiviral immune response [22]. The production of cytokines is an important component of the body’s natural immune response [23,24,25].

It has been shown that SR717 is effective against PRRSV infection in the present study. SR717 stimulates the STING pathway, which induces the expression of antiviral cytokines as a result. In addition to providing novel insights into PRRSV replication inhibition, these findings suggest a new mechanism by which SR717 inhibits PRRSV replication.

## 2. Materials and Methods

### 2.1. Cells, Viruses, Antibodies, and Compounds

African green monkey kidney cells (Marc-145) were meticulously maintained by the esteemed National Joint International Research Center for Animal Immunology at the School of Animal Medicine, Henan Agricultural University. Meanwhile, porcine alveolar macrophages (PAMs) were graciously contributed by none other than Dr. Xuehui Cai, an illustrious research fellow at the Harbin Institute of Veterinary Research affiliated with the prestigious Chinese Academy of Agricultural Sciences. These cell lines have proven to be highly receptive to infection by porcine reproductive and respiratory syndrome virus (PRRSV).

PRRSV-SD16 strain (GenBank: JX087437.1), PRRSV-GD-HD strain (GenBank: EU825724.1), PRRSV-VR2332 strain (GenBank: EF536003.1), PRRSV-JXA1 strain (GenBank: EF112445.1) and PRRSV-CH-1a strain (GenBank: EU273689.1) were given by Professor Enmin Zhou of the Northwest Agriculture and Forestry University of Science and Technology.

In our laboratory, we engineered monoclonal antibodies specific to the mouse anti-N protein. We obtained the Beta Actin Rabbit Receptor Antibody from Proteintech Group (Wuhan, China). Additionally, we procured various antibodies from Cell Signaling Technology (Danvers, MA, USA), including IRF-3 Rabbit monoclonal antibody, P-IRF-3 Rabbit monoclonal antibody, TBK-1 Rabbit monoclonal antibody, P-TBK-1 Rabbit monoclonal antibody, STING Rabbit monoclonal antibody, and P-STING Rabbit monoclonal antibody.

SR717 was kindly presented by Prof. Guoyu Yang of Henan Agricultural University.

### 2.2. CCK8 Assay for Cellular Activity

The concentrations of Marc-145 cells and PAMs were set to 1 × 10^5^ cells/mL. Subsequently, this cell suspension was distributed into 96-well plates, where the cells were incubated for a period of 24 h. SR717 was diluted in 3% FBS/DMEM at 0 µmol/L, 0.6 µmol/L, 2 µmol/L, 6 µmol/L, 20 µmol/L, and treated in the same way with DMSO. The cells underwent an incubation period of four hours, followed by the addition of 10 μL of CCK solution to each well. They continued to incubate for an additional three hours. Subsequently, the absorbance was recorded at 450 nm utilizing an enzyme marker, and the resulting data were analyzed.

### 2.3. Flow Cytometry

A 24-well plate was prepared, and Marc-145 cells were seeded at a concentration of 1 × 10^5^ cells/mL. Once the cells reached 60% confluence, they underwent a four-hour pretreatment phase. Subsequently, the medium was thrown away, and the cells were rinsed with PBS three times. After counting and assessing the cells, they were infected with PRRSV-GFP at a multiplicity of infection (MOI) of 1. This viral exposure lasted only two hours before being gently washed away, and then the cells were supplemented with 1% FBS in DMEM. Flow detection was executed after a 24 h incubation period.

### 2.4. RNA Extraction and Quantitative Real-Time PCR (qPCR)

The Marc-145 cells were introduced with great care into six-well plates, where they settled down at an exact density of one million per milliliter. Once these cells had achieved approximately two-thirds of their potential expansion, we commenced our preliminary treatments. The cells were inoculated with PRRSV-SD16 at a multiplicity of infection (MOI) of 1 for a period of two hours. After discarding the culture medium, we proceeded by introducing 1 mL of Trizol (NCM, Suzhou, China) into the cell sample to disrupt their membranes. Subsequently, 200 μL of chloroform were mixed in, followed by vigorous shaking before subjecting the solution to centrifugation at 15,000 rpm for ten minutes, all performed within a temperature-controlled environment at four degrees Celsius. The supernatant was discarded, 1 mL of alcohol prepared with 75% DEPC water was added, the bottom of the tube was flicked to disperse the precipitate, and the mixture was centrifuged at 15,000 rpm, 4 °C, for 10 min. Following centrifugation, the precipitate was dried at room temperature and DEPC water was added to achieve a concentration of 1 µg/µL. The RNA could then be reverse transcribed into cDNA, with the following preparation of the reverse transcription system being recommended.

Total RNA was isolated from different cell samples utilizing TRNaZol Reagent (NCM, Suzhou, China), following the guidelines provided by the manufacturer. The following components were combined in a 20 μL reaction volume: KEIris×RT All-in-One Mix (4 μL), dsDNase (1 μL), template RNA (1 µg), and RNase-free water. The reaction procedure was 37 °C for 2 min to remove the contaminated DNA from the genome, 55 °C for 15 min, and 85 °C for 5 min to finish the reaction. The cDNA obtained from the reaction was added to DEPC water for 10-fold dilution, and the obtained cDNA was stored in a refrigerator at −20 °C for backup; and, the cDNA was subjected to real-time PCR.

### 2.5. Western Blot Analysis

Marc-145 cells were seeded into six-well plates at a concentration of 1 × 10^5^ cells/mL. Once the cells reached 60% confluence, they were prepped and subjected to the attack phase. When the cell lesions reached 60%, the cells were collected. Western blot analysis (WB) was performed as described previously with the following modifications [26]. Add 100 μL of RIPA (NCM, Suzhou, China) lysis solution, crush the cells. Centrifuge the fragmented cells. Prepare SDS-PAGE gel according to the SDS-PAGE kit, add protein samples to SDS-PAGE gel, and perform electrophoresis. The membrane should be subjected to a wet transfer process for a duration of 80 min at an applied voltage of 110 volts. Following this, the transferred PVDF membrane will be incubated in a solution of 5% skimmed milk for a period of 2 h. The antibody will be incubated after the closure. After incubation, the luminescent solution is to be evenly coated on the PVDF membrane and the membrane, should be placed in a chemiluminescent imager for detection.

### 2.6. Immunofluorescence Assays (IFA)

An immunofluorescence assay (IFA) was performed as described previously, with the following modifications [27]. Marc-145 cells were inoculated onto cell crawlers at a concentration of 1 × 10^5^ cells/mL. When the cells reached 60% confluence, they were prepped and subjected to the previously described procedure. After 36 h, 300 μL of 4% paraformaldehyde (Xilong Science, Shantou, China) was introduced to each well. Subsequently, after an overnight incubation at 4 °C, 300 μL of 0.1% Triton-100 (Solarbio, Beijing, China) was added to each well, and 1% BSA (Solarbio, Beijig, China) (0.5 g + 50 mL PBS) was added after 15 min of action. (50 mL PBS) With total of 300 μL, incubate the antibody after 2 h of action at room temperature and observe under the fluorescence microscope.

### 2.7. Virus Titration

Marc-145 cells were inoculated onto cell crawlers at a concentration of 1 × 10^5^ cells/mL. At 60% growth, the cells were pretreated and attacked using the aforementioned method. Marc-145 cells were inoculated in 96-well plates at a concentration of 1 × 10^5^ cells/mL, and the titer was measured when the cells reached 60% growth. The above viral solution was diluted multiplicatively, the medium was discarded, and 100 μL of the diluted viral solution was added to each well. The cell lesions were observed for 5 consecutive days and the lesions in each well were recorded. The TCID_50_ value of the viral solution to be tested was calculated according to the Karber method [28].

### 2.8. Virus Adsorption Assay

A 6-well plate was selected, and Marc-145 cells were inoculated into the 24-well plate at a concentration of 1 × 10^5^ cells/mL, resulting in a total of four wells. The cells were categorized into two groups. Once the cells had reached 60% confluence, SR717 was diluted in 10% FBS/DMEM medium at a working concentration of 6 µmol/L for a period of four hours. The cells experienced an incubation involving exposure to PRRSV-SD16 at 1 MOI, after which they were chilled at 4 °C for exactly one hour. Afterward, extra fluids were eliminated, and cellular genetic material (RNA) was extracted and reversed into analyzable DNA via PCR methods.

### 2.9. Virus Internalization Assay

A 6-well plate was taken, and Marc-145 cells were inoculated into a 24-well plate at a concentration of 1 × 10^5^ cells/mL. When the cells reached 60%, SR717 was diluted in 10% FBS/DMEM medium at an optimal working concentration of 6 µmol/L and pretreated for 4 h. PRRSV-SD16 was inoculated with Marc-145 cells at 1 MOI, and the cells were placed in a refrigerator at 4 °C for 1 h. After 1 h, the 6-well plates were incubated at 37 °C for 2 h in a 5% CO_2_ incubator. After 2 h, the supernatant was discarded and cellular RNA was collected, reverse transcribed and detected by real-time PCR using the above method.

### 2.10. Virus Replication Assay

A 24-well plate was taken, cell crawlers were placed into the wells, and Marc-145 cells were inoculated onto the cell crawlers at a concentration of 1 × 10^5^ cells/mL. The cells were pretreated with SR717, and diluted at 6 µmol/L in 10% FBS/DMEM medium for a period of four hours. We tested the plates against PRRSV-SD16 at 1 MOI to ensure infection, incubating at 37 °C with 5% CO_2_ for several hours. Following this, we discarded the infected cells and washed the plates thrice with PBS, then transferred to new media containing 1% FBS/DMEM. The cells were examined by IFA at 6 h, 12 h, 24 h, 36 h, and 48 h, respectively.

### 2.11. Virus Assembly Assay

A 6-well plate was selected, and Marc-145 cells were inoculated into a 24-well plate at a concentration of 1 × 10^5^ cells/mL, resulting in a total of six wells. The cells were organized into three distinct groups, designated as groups #1, #2, and #3. When the cells reached 60%, SR717 was diluted in 10% FBS/DMEM medium at an optimal working concentration of 6 µmol/L and the cells were pretreated for 4 h. Marc-145 cells were infected with PRRSV-SD16 at 1 MOI. They were incubated in a CO_2_-rich atmosphere (5%) at 37 °C for 48 h. PBS was used to wash the cells 3 times and 1% FBS/DMEM medium was added. When the observed cytopathic lesions reached 70%, cell supernatant was collected from cell group #1 and the viral genome was extracted.

### 2.12. Virus Release Assay

Marc-145 cells were seeded at a density of 1 × 10^5^ cells/mL in six-well plates and divided into three groups of two wells each. One set received dimethyl sulfoxide as a control, while another was treated with SR717 before viral exposure as per the protocol. When the cell lesions reached 70%, one group repeatedly freeze-thawed the supernatant three times, the second group directly collected the supernatant, and the third group discarded the supernatant and added the same volume of 1% FBS/DMEM and repeatedly freeze-thawed the supernatant three times, and then collected the supernatant. The collected supernatant was subjected to titer determination, and the TCID_50_ values for the viral solution under examination was calculated using the Karber method. The TCID_50_ values derived from this analysis were then compared between the second and third groups and the first group.

### 2.13. Statistical Analysis

Each experiment was meticulously performed three times and then subjected to a statistical review via GraphPad Prism 8 software. The findings are presented as averages along with their respective standard deviations. The statistical significance of the findings was evaluated using Student’s *t*-test, where *p* values below 0.05 (*), 0.01 (**), 0.001 (***) and 0.0001 (****) were acknowledged as indicators of different levels of statistical importance.

## 3. Results

### 3.1. Different Concentrations of SR717 Inhibit the Proliferation of PRRSV

**Figure 1:**
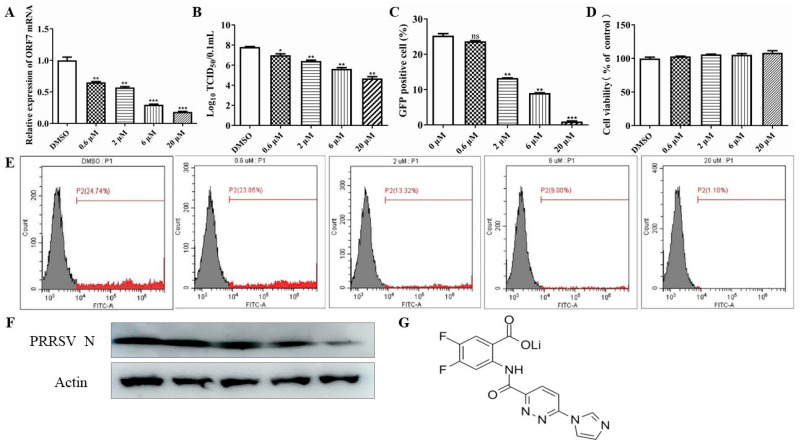
Study on the impact of different concentrations of SR717 on PRRSV. The CCK8 (**D**) assay was employed to assess the impact of varying SR717 concentrations on Marc-145 cell activity at 0, 0.6, 2, 6, and 20 (µmol/L). Flow cytometry (**C**,**E**), Real-time PCR (**A**), Western Blot (**F**), and TCID_50_ (**B**) assay were used to determine the impact of varying concentrations of SR717 on PRRSV-SD16. Chemical structure of SR717 (**G**). SR717 concentrations were 0, 0.6, 2, 6, and 20 (µmol/L); * *p* < 0.05, ** *p* < 0.01, *** *p* < 0.001.

To explore the inhibitory influence of SR717 on PRRSV, we first assessed how different concentrations of SR717 affected the activity of Marc-145 cells by using a CCK-8 assay (Figure 1G). In this study, SR717 was prepared at concentrations of 0 µmol/L, 0.6 µmol/L, 2 µmol/L, 6 µmol/L, and 20 µmol/L. The survival rate of Marc-145 cells across these different SR717 levels appeared similar to that of the control group, indicating that the previously mentioned concentration gradient did not have a noticeable effect (Figure 1D).

Following the pretreatment of cells with SR717, the cells were subsequently infected with PRRSV-GFP at 1 MOI. After 36 h, the supernatant was removed in order to harvest the cells for suspension preparation. The impact of various SR717 concentrations on the growth of PRRSV-infected cells was then assessed using flow cytometry. With a rise in SR717 concentration, the suppression of PRRSV-GFP became more pronounced when contrasted with the control group. Additionally, the viral count, as indicated by fluorescence, diminished in a manner directly related to the concentration levels, as compared to the control group (Figure 1C,E). In addition, supernatant and cell samples from PRRSV-SD16 (MOI = 1) infected for 48 hpi were also collected and assayed for PRRSV ORF7 gene expression (Figure 1A), viral titer (Figure 1B), and viral N protein expression (Figure 1F). The results demonstrated that SR717 exhibited a notable inhibitory impact on PRRSV infection, with a concentration gradient-dependent effect.

### 3.2. Inhibitory Effect of SR717 on Marc-145 Cells Infected with Different PRRSV Strains

**Figure 2:**
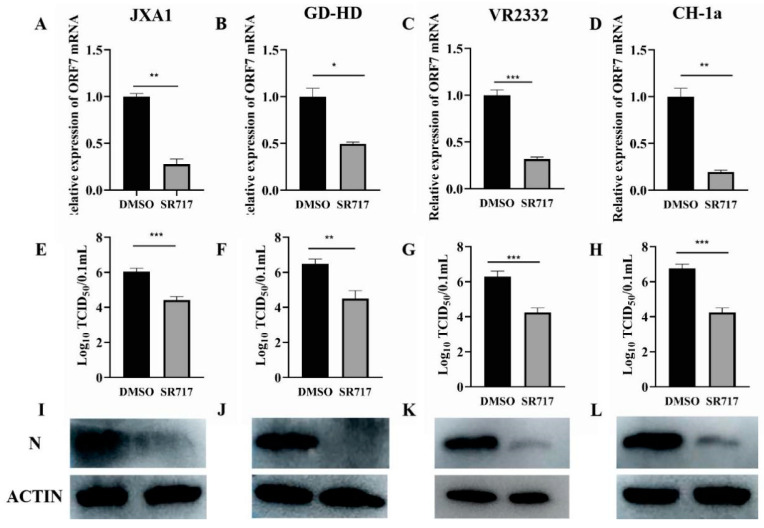
Study of the effect of SR717 on different PRRSV strains. Marc-145 cells were inoculated in 6-well plates and cultured for 12 h prior to being pretreated with SR717. Following infection with four distinct PRRSV strains (JXA1, GD-HD, VR2332, and CH-1a), supernatants and cell samples were collected for the purpose of detecting the expression level of the PRRSV ORF7 gene, viral titer, and PRRSV N protein expression. (**A**,**E**,**I**): PRRSV-JXA1 strain; (**B**,**F**,**J**): PRRSV-GD-HD strain; (**C**,**G**,**K**): PRRSV-VR2332 strain; (**D**,**H**,**L**): PRRSV-CH-1a strain; * *p* < 0.05, ** *p* < 0.01, *** *p* < 0.001.

To delve deeper into the impact of SR717 on PRRSV, we exposed Marc-145 cells (6 μmol/L) to various PRRSV strains (JXA1, GD-HD, VR2332, and CH-1a) according to an MOI of 1, and examined the expression level of the PRRSV ORF7 gene with selected concentrations at 36 h; SR717 treatment significantly reduced the replication of the assorted PRRSV strains (Figure 2A–D). To corroborate the inhibitory effects of SR717 on various strains of PRRSV, we exposed Marc-145 cells that had been treated with SR717 to different PRRSV strains. After 48 h, we harvested the liquid portion above the cell layer known as the “supernatant”, along with the actual cells themselves to measure the virus concentration (Figure 2E–H) and evaluate the presence of the PRRSV nucleocapsid protein (Figure 2I–L). The findings indicated that SR717 inhibited PRRSV proliferation without strain specificity.

### 3.3. Inhibitory Effect of SR717 on PAMs Infected with Different PRRSV Strains

**Figure 3:**
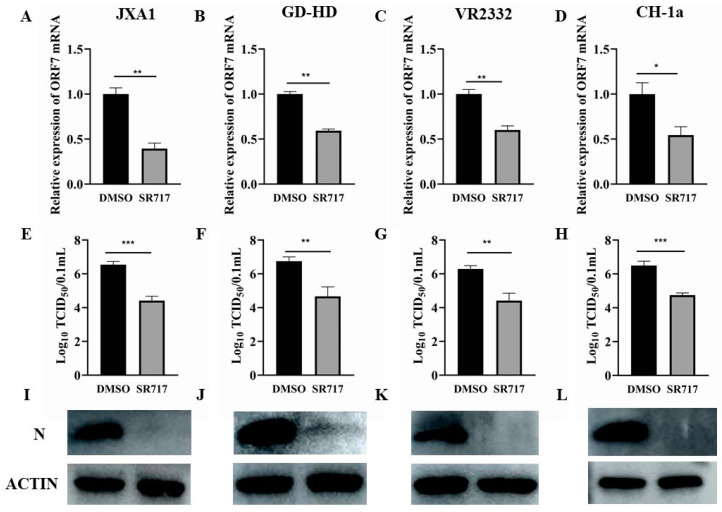
Inhibitory effect of SR717 on PAMs infected with different PRRSV strains. PAMs were inoculated in 6-well plates, cultured for 12 h and pretreated with SR717, and affected with different PRRSV strains (JXA1, GD-HD, VR2332, and CH-1a). Supernatants and cell samples were collected for the purpose of detecting the expression level of the PRRSV ORF7 gene, viral titer and PRRSV N protein expression. (**A**,**E**,**I**): PRRSV-JXA1 strain; (**B**,**F**,**J**): PRRSV-GD-HD strain; (**C**,**G**,**K**): PRRSV-VR2332 strain; (**D**,**H**,**L**): PRRSV-CH-1a strain; * *p* < 0.05, ** *p* < 0.01, *** *p* < 0.001.

To evaluate the inhibitory effect of SR717 on the infection of different virulent strains of PRRSV in PAMs, PAMs were inoculated in 6-well plates and cultured for 12 h and pretreated with SR717, and infected with different virulent strains of PRRSV (JXA1, GD-HD, VR2332, and CH-1a) for 36 h. Cells and fluids were gathered, and PRRSV factors were examined, including ORF7 gene expression (Figure 3A–D), viral load (Figure 3E–H), and nucleocapsid protein production as an infection marker (Figure 3I–L). Data in Figure 3 demonstrates SR717’s ability to inhibit both ORF7 expression and PRRSV’s N protein of PRRSV-JXA1 strain, PRRSV-GD-HD strain, PRRSV-VR2332 strain, and PRRSV-CH-1a strain with a significant effect after acting on PAMs.

### 3.4. SR717 Blocks PRRSV Genome Replication, Assembly, and Release, but Has No Effect on Virus Adsorption and Entry

**Figure 4:**
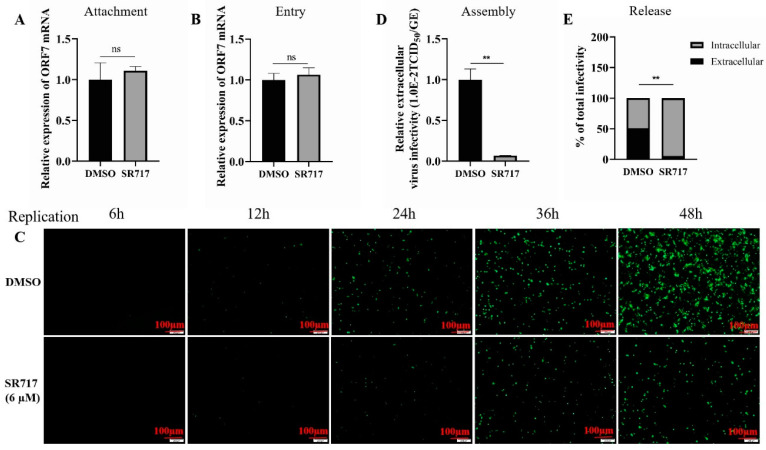
Study on the effect of SR717 on the life cycle of PRRSV. (**A**) Real-time PCR to detect the effect of SR717 (6 µmol/L) on the adsorption phase of PRRSV following its administration to Marc-145 cells. (**B**) Real-time PCR was performed to detect the effect of SR717 (6 µmol/L) on PRRSV’s entry phase after acting on Marc-145 cells. (**C**) IFA assay for the effect of SR717 (6 µmol/L) on PRRSV replication phase after acting on Marc-145 cells; (**D**) to ascertain the impact of SR717 (6 µmol/L) on the assembly phase of PRRSV following its administration to Marc-145 cells; (**E**) to detect the effect of SR717 (6 µmol/L) on the release phase of PRRSV subsequent to its action on Marc-145 cells; ** *p* < 0.01.

To gain further insight into the viral life cycle that is inhibited by SR717, a series of experiments were conducted, including those investigating adsorption, entry, replication, assembly, and release (Figure 4). In the adsorption study, Marc-145 cells were treated with SR717 at a concentration of 6 µmol/L and subsequently infected with PRRSV at 1 MOI. The cells were then incubated at 4 °C for 2 h to facilitate the attachment of the virus. After this incubation period, cellular RNA was extracted and underwent reverse transcription. Real-time PCR was employed to ascertain the expression of the PRRSV ORF7 gene. No significant disparity in gene expression was observed between the two groups during the period of virus adsorption (Figure 4A). Upon completion of the adsorption phase, the cells were incubated at an ambient temperature of 37 degrees Celsius for two hours, allowing sufficient time for viral penetration and internalization. These findings are graphically represented in Figure 4B. The expression levels of the PRRSV ORF7 gene in the Marc-145 cell group treated with 6 µmol/L SR717 did not show any notable differences when compared to the control group. In replication experiments, cells were treated with SR717 (6 µmol/L) for 4 h and inoculated with PRRSV at 1 MOI. The results are shown in Figure 4C. After 6 and 12 h, fluorescing particle quantities did not differ substantially between treated cells (SR717) and controls; however, after 24, 36, and 48 h, there was a significant decrease in these particles compared to the control group.

In the assembly experiment, Marc-145 cells were seeded and subsequently infected with PRRSV at a multiplicity of infection of 1, using SR717 at a concentration of 6 µmol/L, for a duration of 4 h. After a period of 36 h, the efficiency of the virus assembly was assessed by analyzing the infection titer (TCID_50_/mL) alongside the total genomic equivalent of PRRSV, with the findings illustrated in Figure 4D. In release assays, Marc-145 cells that had reached normal viral replication after 8 h of inoculation with PRRSV were treated with SR717 (6 μmol/L). After 24 h, intracellular and extracellular infectious viral particles were measured by the TCID_50_ assay. The ratio of extracellular virus titer to total virus titer indicated the percentage of virus release. The percentage of viral release was found to be lower in the SR717-treated cell group than in the control group (Figure 4E).

### 3.5. SR717 Treatment Promotes STING-Mediated Expression of Type I Interferon and Antiviral Cytokines

**Figure 5:**
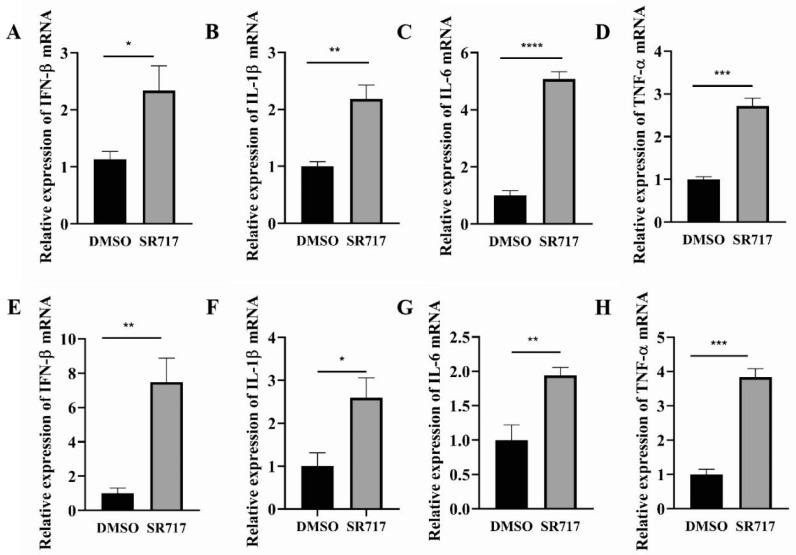
SR717 treatment promoted STING-mediated expression of type I interferon and proinflammatory cytokines. Real-time PCR assay of cytokine production after SR717 action on Marc-145 cells (**A**–**D**) and PAMs (**E**–**H**) and PRRSV infection; * *p* < 0.05, ** *p* < 0.01, *** *p* < 0.001, **** *p* < 0.0001.

To examine the antiviral mechanism of SR717, we utilized real-time PCR to measure cytokine production after treating Marc-145 cells and PAMs infected with PRRSV. Figure 5 showcases the outcomes after SR717 treatment has been applied to both Marc-145 cells and PAMs; there was a noticeable increase in the expression levels of the IFN-β, IL-1β, IL-6, and TNF-α genes when compared to the control group. Thus, the study revealed that SR717 effectively countered PRRSV infections in both Marc-145 cell cultures and PAMs through its ability to stimulate the production of key immune response mediators such as IFN-β, IL-1β, IL-6, and TNF-α.

### 3.6. SR717 Can Activate the STING Pathway to Induce the Expression of Antiviral Cytokines

**Figure 6:**
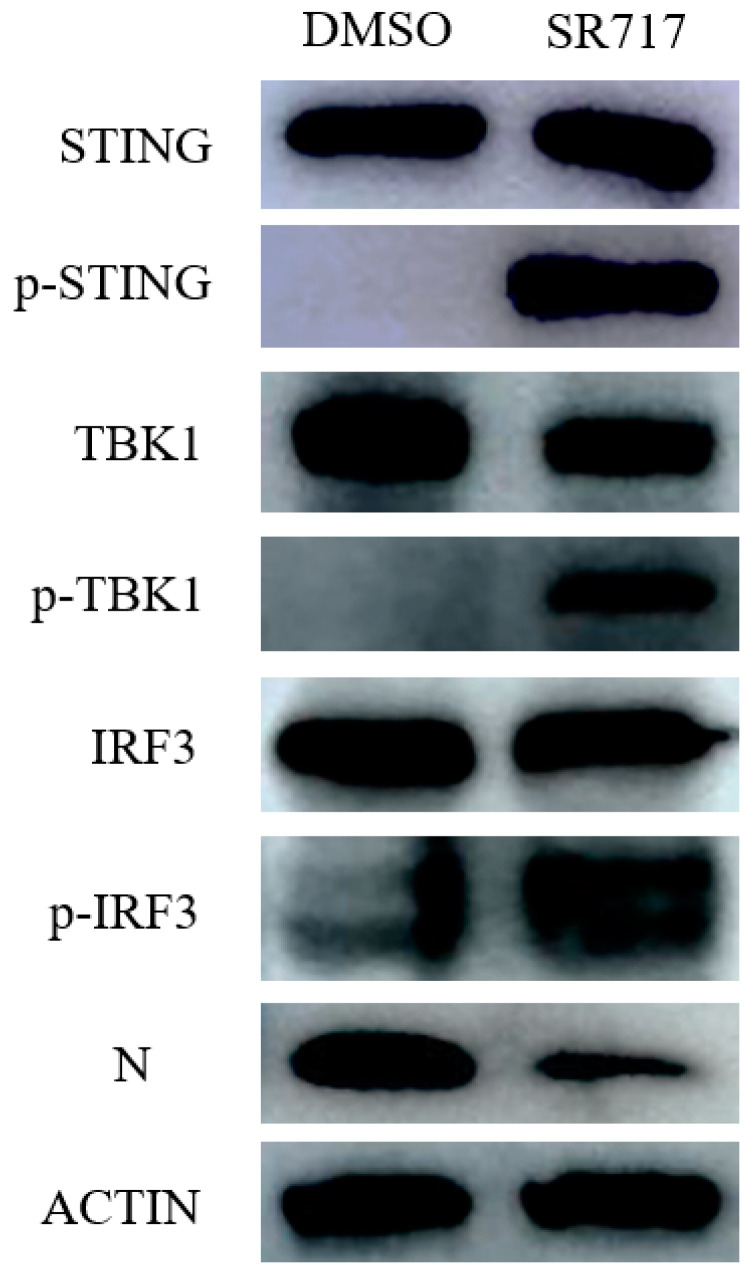
Study on the effect of SR717 on the life cycle of PRRSV. PRRSV inhibits STING-mediated downstream pathways. Marc-145 cells were treated with SR717 and infected with PRRSV (MOI = 1) for 48 h. The cells were harvested and analyzed through Western blotting using antibodies targeting phosphorylated TBK1 (p-TBK1), TBK1, phosphorylated IRF3 (p-IRF3), IRF3, PRRSV-N, and β-actin.

As shown earlier, SR717 influences PRRSV-infected Marc-145 cells and PAMs, promoting the production of IFN-β, IL-1β, IL-6, and TNF-α. In order to further investigate SR717’s antiviral mechanism, we employed the Western blot method to examine the expression levels of key proteins in the STING pathway after treating Marc-145 cells infected with the PRRSV-SD16 strain using SR717. The outcomes are depicted in Figure 6. In contrast with the control group, SR717 induced the phosphorylation of STING, TBK1, and IRF3 proteins after the action of SR717 on Marc-145, indicating that SR717 can activate the STING pathway, thereby inducing the expression of antiviral cytokines and exert antiviral effects.

## 4. Discussion

The rapid mutation of the PRRSV genome, the high frequency of new strains and the immune evasion characteristics of PRRSV result in limited protection being provided by vaccines. Moreover, current commercial vaccines do not offer lasting protection and management against PRRSV. This has led to considerable difficulties in preventing and controlling PRRSV, with the disease remaining challenging to eradicate globally. As a natural compound, SR717 can effectively circumvent the risk of reversion to virulence while offering a cost-effective and safer alternative to vaccines, providing a novel avenue for the prevention and control of PRRSV [29]. SR717 is a non-nucleotide analogue of cGAMP, derived from its structural design. STING proteins are transmembrane proteins located in the endoplasmic reticulum, and STING is an important signaling protein associated with the natural immune system [30].

Several studies have demonstrated that STING is capable of inhibiting the proliferation of DNA viruses by mediating the production of cytokines, while being able to inhibit RNA virus proliferation [31,32]. Cells knocked down to express STING inhibited the production of inflammatory factors after infection with Sendai virus, and STING knockout mice were able to respond significantly to RNA viruses; additionally, RNA viruses were able to replicate in large numbers in STING-knockout cells [33,34]. STING proteins are highly conserved in different organisms. When STING receives signals and is activated, it can activate TBK1 protein, and the activated TBK1 protein can phosphorylate STING proteins, and the phosphorylated STING proteins can increase the affinity for IRF3 proteins, and the phosphorylated IRF3 protein can be activated by the phosphorylation of IRF3 proteins by the interaction of TBK1 and IRF3 proteins [35,36]. TBK1 phosphorylates IRF3 protein, and the phosphorylated IRF3 protein can be activated to produce cytokines to produce antiviral effects [37].

The effect of SR717 on PRRSV was not elucidated in these studies. In this research, we showed that varying concentrations of SR717—specifically 0 µmol/L, 0.6 µmol/L, 2 µmol/L, 6 µmol/L, and 20 µmol/L—effectively suppressed the expression of the PRRSV ORF7 gene, reduced the levels of the PRRSV N protein, decreased progeny viral titers, and inhibited PRRSV replication in both Marc-145 cells and PAMs. This inhibitory effect was significant and exhibited a clear dose-dependent relationship; and, it was found that SR717 at 6 µmol/L was able to inhibit and that 6 µmol/L of SR717 could inhibit the expression of PRRSV-GD-HD, PRRSV-JXA1, PRRSV-VR2332, PRRSV-CH-1a ORF7 gene, and N protein, which was not strain-specific. To rule out the chance that the inhibition of PRRSV by SR717 was due to the cytotoxic effects of SR717 on the cells, the cellular activity of SR717 was examined by CCK8 assay at different concentrations, and the results showed that the above-mentioned concentration gradients of SR717 on Marc-145 cells had no effect on the cellular activity.

To gain deeper insight into how exactly SR717 hinders PRRSV’s ability to proliferate, we probed the influence of SR717 (a concentration of six micromoles per liter) across various phases within the virus’s reproductive process throughout our study. The results demonstrated that SR717 impeded PRRSV proliferation by suppressing the assembly, replication, and release phases of the virus. Furthermore, SR717 administration within Marc-145 cell cultures resulted in an intensification of the phosphorylation process involving STING, TBK1, and IRF3 molecules. This intervention resulted in an upsurge in the expression of the IFN-β, IL-1β, IL-6, and TNF-α genes, subsequently triggering a natural immune response. In conclusion, our research indicates that SR717 has the ability to stimulate the STING pathway, leading to the upregulation of antiviral cytokines that contribute to its antiviral effects. This lays the groundwork for additional investigation into the underlying mechanisms by which SR717 operates.

It remains unclear whether SR717 has a therapeutic effect on pigs infected with PRRSV, despite its anti-PRRSV activity in vitro. To begin with, the safety of SR717 at the level of the organism remains a topic of ongoing discussion. Additionally, the required dosage for inhibiting PRRSV in living organisms, as well as the threshold for toxicity in animals, has yet to be established. Consequently, further investigation is required to ascertain the prophylactic and therapeutic effects of SR717 on PRRSV in vivo.

## Data Availability

All data supporting our findings is contained within the manuscript.
